# Immobilization effects of co-pyrolyzed neem seed mixed with poultry manure on potentially toxic elements in soil and the phytoremediation potentials of native Manihot esculenta and Jatropha curcas in ensuring sustainable land use

**DOI:** 10.1007/s10661-023-11430-3

**Published:** 2023-06-01

**Authors:** Martin Kofi Mensah, Carsten Drebenstedt, Ibukun Momoriola Ola, Nils Hoth, Frederick Gyasi Damptey, Edward Debrah Wiafe

**Affiliations:** 1 Institute of Surface Mining and Special Civil Engineering, Technical University of Mining Freiberg, Gustav-Zeuner Street 1A, 09599 Freiberg, Germany; 2 Department of Ecology, Brandenburg University of Technology, 03046 Cottbus, Germany; 3 School of Natural and Environmental Sciences, University of Environment and Sustainable Development, PMB Somanya, Ghana

**Keywords:** Potentially toxic elements (PTEs), Phytoremediation, Plants, Bioaccumulation, Growth stress

## Abstract

This study evaluated the effects of neem seed biochar, poultry manure, and their combinations at varying rates of 15 and 25% (w/w) on potentially toxic elements (PTEs) in soils. Afterward, the suitability of *Manihot esculenta* and *Jatropha curcas* in removing Cd, As, Zn, Pb, and Hg from mine spoils were appraised in a 270-day outdoor pot experiment. Using ICP–Mass Spectrometry, the elemental contents of target PTE in the shoot, root, and soil specimens were determined for each treatment. The obtained average values were further subjected to a nonparametric test of samples using IBM SPSS Statistic 29. The applied organic amendments resulted in significant differences *p* < 0.05 in PTE availability for plant uptake after the Independent-Samples Kruskal–Wallis Test was made. Nonetheless, applying a 25% (w/w) mixture of neem seed biochar and poultry manure was efficient in immobilizing more PTEs in soils which caused lower PTEs presence in plants. Organic amendments further significantly enhanced the fertility of the mine soils leading to about a 6– 25.00% increase in the biomass yield (*p* < 0.05) of both plants. No significant difference (*p* > 0.05) was however observed between the phytoremediation potentials of both plants after the Independent-Sample Mann–Whitney U test. Even that, *Manihot esculenta* was averagely more efficient in PTE uptake than *Jatropha curcas*. Larger portions of the bioaccumulated PTEs were stored in the roots of both plants leading to high bioconcentration factors of 1.94– 2.47 mg/kg and 1.27– 4.70 mg/kg, respectively, for *Jatropha curcas* and *Manihot esculenta.* A transfer factor < 1 was achieved for all PTEs uptake by both plants and indicated their suitability for phytostabilization*.* Techniques for easy cultivation of root-storing PTEs are required to enhance their large-scale use as their biomass could further be used in clean energy production.

## Introduction

Natural and anthropogenically induced potentially toxic elements “PTEs”﻿ may be metals or metalloids. When they enter our ecosystem, they exhibit the ability to cause toxicity to living organisms even at lower concentrations or when they exceed their permissible environmental limits (Bacchetta et al., 2015; Chen et al., 2022; Dorleku et al., 2018; A. K. Mensah, 2021; Pathak & Shah, 2021; Piršelová, 2011; G. Tóth et al., 2016a, 2016b; Gergely Tóth et al., 2016a, 2016b). Toxic elements usually have a specific gravity of > 5 g/cm^3^ (R. Singh et al., 2018)**.** Their presence and persistence in the environment through industrial, pharmaceutical, agrochemical and mining keep increasing their health risk levels in humans (Antoniadis et al., 2017; Bundschuh et al., 2021; Cordy et al., 2011; Shen et al., 2019; Wiche et al., 2017). Cases of health and safety defects due to human exposures to PTEs remain well documented and include, silicosis, skin defects, pnuemonocosis, and respiratory and heart-related diseases (Antoniadis et al., 2017; Bortey-Sam et al., 2015; Mensah et al., 2020a, 2023)**. ﻿**Others may include reduced fertility in humans, mental retardation, malfunctioning of vital organs, and birth defects (Apostoli & Catalani, 2015; Grotto et al., 2010)**.** Potentially toxic elements are also able to reduce the normal functioning of a plant’s physiological and metabolic activities. Thus, resulting in undesirable growth, diseases, and low yields of arable and tree crops. Their association with gold and other metallic minerals have increased their numerous human risk concerns in Ghana (Mensah et al., 2020a; Mensah et al., 2022a).

Many remediation options for PTEs-laden media exist. These may be physical (dig and wash, membrane treatments, oxide lining, containment), biological treatments (bioremediation with plants and bacteria) and chemical (chelators) (Mehes et al., 2013). However, the application of either option has further implications on cost, soil structure and quality and feasibility in application in large areas. Presently, phytoremediation has proven to be an eco-friendly and cost-effective approach to the other treatment options (Kobina et al., 2021; Mahar et al., 2016; Mensah et al., 2022a; Nwaichi et al., 2009; Okoroafor et al., 2022b). Several phytoremediation techniques such as phytoextraction, phytostabilization, phytofiltration, phytodegradation, phytovolatilization, and rhizodegradation exist (Mehes et al., 2013; Singh et al., 2018). However, their applicability is dependent on the goal of the planned soil remediation programme, as various plants have special attributes for adaptability and efficacy. Nonetheless, the deployment of well-adaptive indigenous hyperaccumulators remains critical for enhanced success rates in the phytoremediation (Antoniadis et al., 2017; Kobina et al., 2021).

Exploring phytoremediation, which is relatively inexpensive and easy to implement presents a more sustainable option in the Ghanaian context. Studies on plant species such as C*hromolaena odorata, Pityrogramma calomelanos, Alchornea cordifolia, Lantana camara* and *Pueraria montana* have been reported in the literature for their PTEs bioaccumulation abilities in Ghana (Bansah et al., 2016; Kobina et al., 2021). However, several limitations existed as most were rather found to have grown or held contents of PTEs in contaminated sites. In such cases, the prevailing baseline physicochemical properties of the soils, growth conditions, and duration for bioaccumulation were not adequately known. In addition, most woody bioaccumulators are reported to take several years before they can significantly clean up contaminated media. A situation that tends to gravely limit the fortunes of phytoremediation, unless assisted with chemical chelators (Okoroafor et al., 2022a). This means a huge knowledge gap still exists on potential accumulator plants and their management in the Ghanaian ecosystems.

The production of biochar is involved the controlled burning of agricultural biomass wastes into activated carbon in the absence of an oxygen (Leng et al., 2022; Zhang et al., 2013). Biochar usually has porous and large surface areas and is alkaline in pH. They are documented to be effective in sustainable soil management through the increase in soil carbon stocks after the sequestration of carbon from the atmosphere, the immobilization of soil contaminants, and the improvement of soil nutrition and health (Lu et al., 2017). Thus, their addition to soils results in soil organic carbon (SOC), pH, and macro and micro-nutrient increases (Amirahmadi et al., 2020). Similarly, organic amendments which can be obtained from either flora or fauna (e.g., bird droppings, mulch) are eco-friendly substances capable of maintaining the integrity of soils through nutrient supply and the reduction of soil toxicity from metals and metalloids (Amirahmadi et al., 2020).

This study aimed at evaluating the effect of selected organic amendments on soil contaminants and how.indigenous shrubs could efficiently remove PTEs in contaminated gold mine spoils under growth stress conditions. Apart from the conventional criteria for selecting plants for phytoremediation, considerations were given to the possible beneficial reuse of the biomass generated without causing health and safety concerns. This is an approach that could help explore future self-financing models through participatory approaches by local communities. As much as we are aware, the bioremediation potentials of neem seed biochar and *M. esculenta* on multi-contaminated soils have not been explicitly explored in science.

## Materials and Methods

### Plant selection

Two (2) local plant species, *Jatropha curcas* and *Manihot esculenta* were used as test plants in this study. Before the main experiment, *J*. *curcas* was raised by seeds and nursed until 8 weeks old and at an average plant height of 10.3 cm. Whilst *M*. *esculenta* stem, cuttings were pre-sprouted under moist conditions only (without soil) for 21 days before planting. Plant selection was based on more than one of the following attributes: their ability to withstand harsh growing conditions, moderate lifespan, potential to bioaccumulate soil PTEs, fast biomass growth, ability to grow on poor soils, prudent root structures, and the potential reusability of contaminated biomass amongst others.

### Pot preparations and experimental design

Bulk soil samples (Table 1) from an oxide gold mine spoil in Southern Ghana described in our previous study (Mensah et al., 2023) were used. The prepared soil treatments in pots were made to stand for 21 days before planting but were watered two times per week. Before planting, portions of the soils were augmented with organic amendments at different combinations and application rates (w/w). The treatments included the addition of 15% poultry manure (15% PM), 25% poultry manure (25% PM), 15% neem seed biochar (15% NE), 25% activated neem seed extract (25% NE), 15% of mixed neem seed biochar and poultry manure (15% NE + PM), 25% of mixed neem seed biochar and poultry manure (25% NE + PM) to the reference soils. The contaminated mine soil alone was used as control. Thus, a total of seven soil treatments were investigated. A proximate analysis of the used poultry manure and neem seed biochar is presented in Table 2 after using a PerkinElmer 2400 elemental analyzer.

**Table 1:** Physicochemical characteristics of sample soils

Parameters	Unit	Value
Silt	(%)	50.50
Clay	(%)	26.00
Sand	(%)	13.50
Loam	(%)	-
Gravel	(%)	10.00
Field capacity	(%)	55.00
Bulk density	g/cm^3^	1.83
Cadmium (Cd)	mg/ks	28.52
Arsenic (As)	mg/kg	326.17
Lead (Pb)	mg/kg	30.08
Mercury (Hg)	mg/kg	7.60
Zinc (Zn)	mg/kg	261.39
Soil Carbon	mg/kg	0.90
Total Nitrogen (N)	(%)	0.08
Phosphorous	mg/kg	3.82
pH	-	4.33
Electrical conductivity (EC)	(μs/cm)	1970.00
Magnesium (Mg)	(cmol/ kg)	0.40
Calcium (Ca)	(cmol/ kg)	2.20
Potassium (K)	(cmol/ kg)	0.09

**Table 2:** Proximate analysis of poultry manure and activated neem seed (biochar)

Parameter	Poultry Manure		Activated Neem Extract
	Content	Unit	Content
Nitrogen	0.81	%	0.36
Phosphorus	162.05	mg/kg	109.00
Potassium	153.00	mg/kg	406.30
Organic carbon	122.40	mg/kg	281.60
Sulphur	4.90	mg/kg	-
Calcium	5.10	cmol/ kg	6.09
Magnesium ﻿	2.60	cmol/ kg	1.63
C/N ratio	﻿3.72	%	101.7
EC	585	μs/cm	308
pH	7.60	-	8.42
CEC	-	cmol/ kg	﻿16.19

Essential treatment preparations before incorporation into soils were made in advance. For the neem seed biochar, ripened neem seeds were gathered, soaked in water for 72 h, fruits removed, and air-dried for one week. Later, seed activation was done in a furnace at 400 degrees for 1.5 h without oxygen. For the poultry manure, eight weeks old bird litter was sterilized at 105 degrees for 48 h and cooled before the treatment combinations were made. Each amendment was mixed with 12 kg of contaminated soil and filled in a 12-L-pot size. The experiment was laid in a randomised complete block design (RCBD), with three plants per stand and three replications each. Thinning to two plants per stand was done after 90 DAP. The setup was done outdoors to mimic natural field conditions.

### Agronomic Management

After transplanting, a thrice-a-week watering regime at 75% field capacity was done for 30 days until all plants had stabilized. Later, one week of watering still at 75% field capacity was maintained throughout the study unless the incidence of rains occurred. Plants were exposed to natural weed competitions and controlled at 80, 170 and 260 days after planting (DAP). No pest or disease control was done throughout this study. Basic agronomic care and monitoring were done routinely and documented throughout the study. The average temperature and humidity during the study were monitored. Growth parameters (plant height, number of leaves, root length, and biomass weight) per plant were measured during biomass harvesting.

### Biomass and soil PTE analysis

Plant biomass (shoot and root parts) and soil samples were harvested after 270 DAP. Some weeds that grew in some pots were randomly sampled and analyzed. At harvesting, care was taken to cause fewer or no injuries to the roots. Where roots were lost to the soil, their debris was sufficiently reclaimed. Harvested specimens were washed under running water to remove all soil particles and later rinsed with deionised water. Initial parameters (fresh weight and length) were measured, air-dried, oven-dried at 75 °C for 48 h, cooled, weighed, and milled with a centrifugal mill (ZM1000, Retsch, Germany). Sample weights of 100.00 mg each were microwave digested with Ethos plus 2, MLS, as described by Okoroafor et al., (2022a). The digestion was aided by 3.00 mL nitric acid and 0.10 mL hydrofluoric acid. The total concentration of the obtained extracts was measured using ICP-MS (Xseries 2, Thermo Scientific). The elemental value obtained per sample was multiplied by the corresponding dry biomass weight per plant. For the elemental contents in soils, the same methods were used in our previous study (Mensah et al., 2023). The target PTE investigated were contents of As, Cd, Hg, Pb and Zn in the shoot and roots of both plants and their growth media.

### Estimation of phytoremediation potentials of test plants

The movement of PTEs from soil to plant indices was used to ascertain the suitability of the selected test plants for remediation after the experiment. This involved estimating how much either the roots or shoots of each plant had stored PTE from their growth media through the estimation of bioconcentration factor (BCF) in Eq. 1 or bioaccumulation factor (BAF) in Eq. 2 relative to the post-remediation soil PTE contents (Kobina et al., 2021; Mensah et al., 2020b).

1 $$BCF=PTE\;in\frac{Root}{soil} (mg/kg)$$

2 $$BAF=PTE\;in\frac{Shoot}{soil} (mg/kg)$$

For each plant to qualify as an accumulator, it must either have BCF or BAF values higher than 1. Therefore, an estimation of translocation factor (TF) using Eq. 3 helped to determine the test plants' ability to transport soil PTEs into shoot biomass.

3 $$TF=PTE\;in\frac{Shoot}{Root} (mg/kg)$$

A plant with a BAF and TF > 1 may be classified as a suitable specimen for phytoextraction. However, when a plant has a BCF > 1 < TF, they exhibit phytostabilizering potentials (Kobina et al., 2021; Mehes et al., 2013; Mensah et al., 2022a; Mishra & Pandey, 2018).

### Data treatments and analysis

Using IBM SPSS Statistic 29, a nonparametric test of treatment was made at a confidence level of 95%. Thus, the phytoremediation performance of both *J. curcas* and *M. esculenta* was analyzed using the Independent-Sample Mann–Whitney U test. The effects of the organic amendment application on PTE uptake by plants were analyzed through the Independent-Samples Kruskal–Wallis Test. A pairwise comparison of treatments was also done to determine the variances between each treatment. Using OriginPro 2022b software, bar plots were created, which showcased the mean, median, and outliers. The error bars represent the mean ± standard deviation of the samples analyzed.

## Results

### Growth and climatic information

Table 1 provides background data on the polluted mine soil used for the experiment. The trace element contents in the soil were higher than the plant-available nutrient levels. The trial's average temperature and relative humidity were 34.1 °C and 72.8%, respectively. Also, the chemical makeup of the neem seed biochar and poultry manure after proximate analysis is presented in Table 2.

Potentially toxic element uptake capacities by test plants.

### Effect of applied amendments on soil PTE and Jatropha curcas remediation performance

**Fig. 1:**
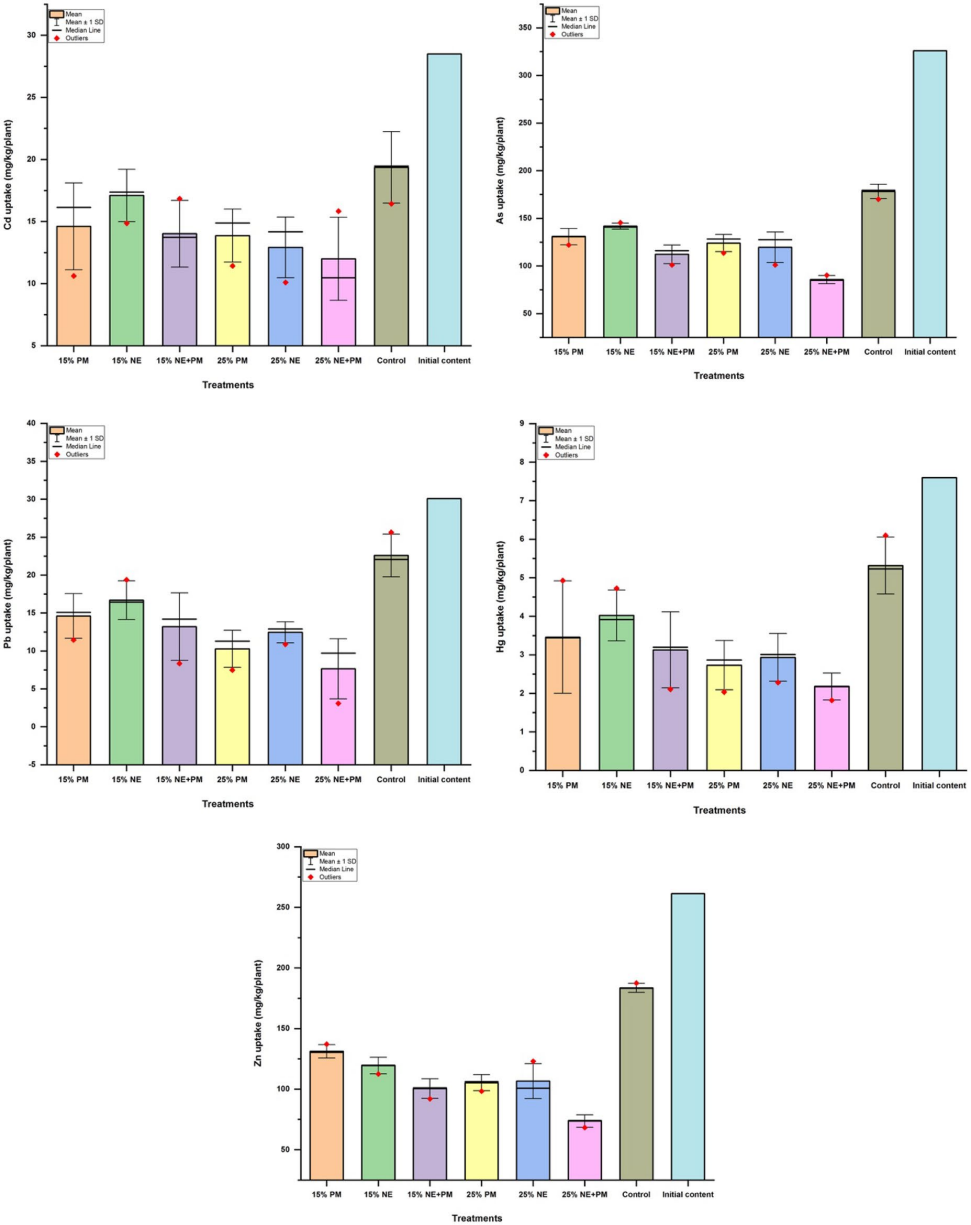
Effects of applied organic amendments on the PTE uptake potentials of *Jatropha. curcas*

The uptake of target PTEs was observed to have mainly occurred in the roots of *J. curcas* after 270 days after planting (DAP). Based on average performances different soil treatments resulted in peculiar outcomes when compared to the control treatments (Fig. 1). Thus, a significant difference (*p* < 0.05) in the reduction of PTE movement from soil to plants in the range (min–max) of 17- 65, 23- 59, and 31- 64.00% for As, Pb and Zn, respectively, was achieved. However, the same amendments did not result in significant differences in Cd and Hg limitation to the *J. curcas* plant even though a reduction range (min–max) of 15- 25 and 26- 59.00%, when compared with the controls, was achieved. Cumulatively, the application of 25% PM + NE was superior to the remaining amendments in limiting As, Pb and Zn uptake from soils by the roots of *J. curcas* roots. Whilst the 25% PM was comparably better in Cd and Hg containment. The application of 15% PM and 15% NE was found to be the least in reducing PTE uptake from soil.

### Effect of applied amendments on Manihot esculenta remediation performance

**Fig. 2:**
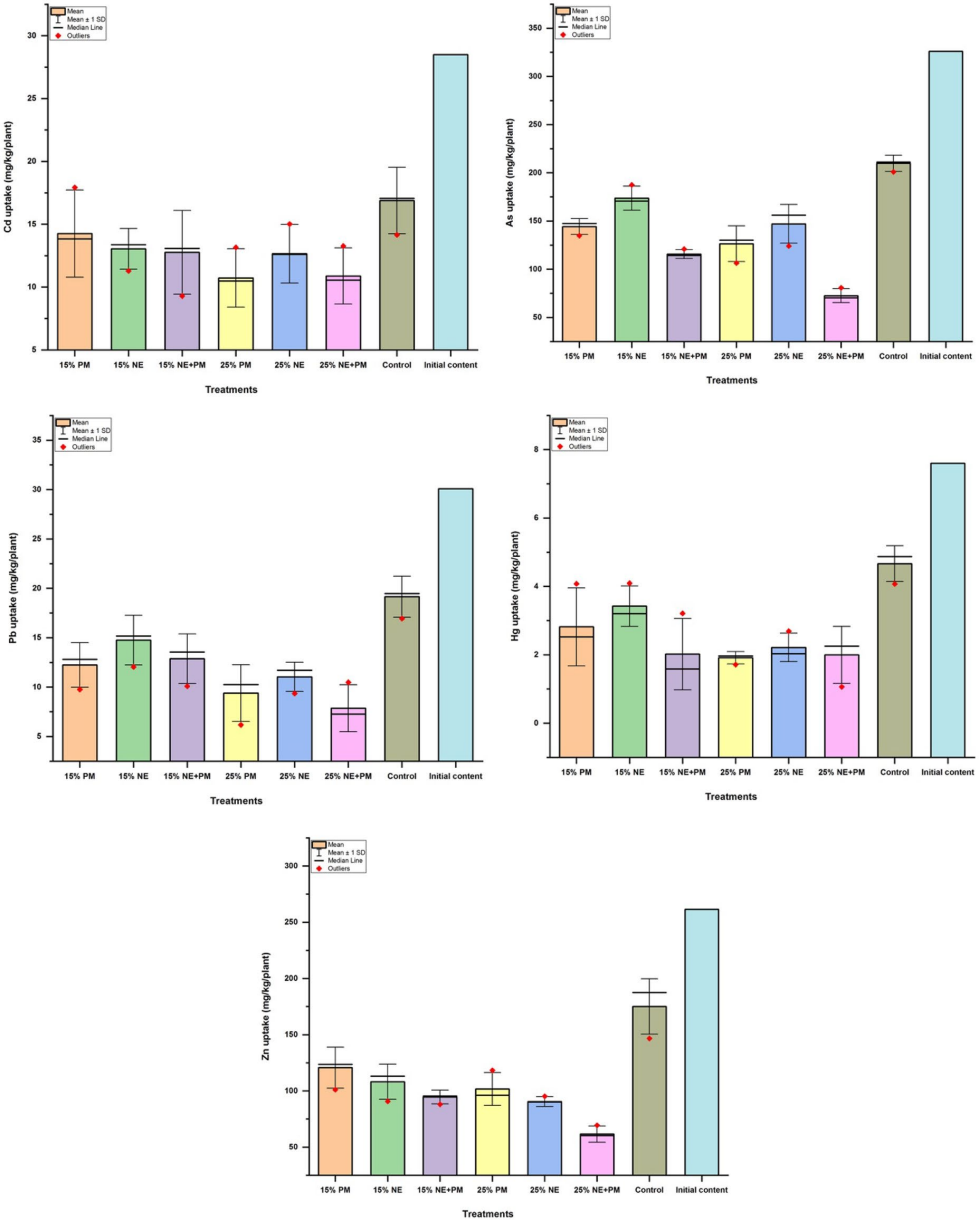
Effects of different soil amendments on the PTE uptake potentials of *Manihot esculenta*

The ground structures of *Manihot esculenta* were able to hold larger portions of the bioaccumulated PTEs from their growth media. The addition of organic amendments and their interaction with *M. esculenta* exhibited similar trends as observed for *J. curcas.* Thus, based on average performances, the observed organic amendments -plant- soil PTE interaction resulted in 11- 38, 20- 52, 26- 66, 24- 59, and 28- 60.00% reduction in the transfer of Cd, As, Pb, Hg and Zn, respectively (Fig. 2). Despite this, the applied amendments were not significant (*p* > 0.05) in limiting the availability of Cd and Hg to *M. esculenta* for uptake. The application of 25% PM + NE was found to be better in limiting the bioavailability of PTEs for plant uptake as compared to the addition of 15% PM or 15% NE to the soil. The pH of the growth media increased on average from 4.8 to a range of 6.70- 8.30.

### Phytoremediation performance of *Jatropha curcas* and *Manihot esculenta*

The general performance of both *J. curcas* and *M. esculenta* in removing the target contaminants from soils under natural conditions was similar (*p* > 0.05). Despite this, average bioaccumulation performances showed that *M. esculenta* was a better accumulator of most PTEs when under natural conditions (Table 3).

**Table 3:** Phytoremediation potentials of *Jatropha curcas* and *Manihot esculenta* under natural conditions

Potentially Toxic Element (PTE)	*Background Content* (mg/kg)	Bioaccumulation (Mean + SD)	
		*Jatropha curcas*	*Manihot esculenta*
Cd	28.50	16.89 ± 2.65	19.36 ± 2.88
As	326.10	209.86 ± 8.38	178.19 ± 7.53
Pb	30.08	19.16 ± 2.08	22.59 ± 2.81
Hg	7.60	4.67 ± 0.52	5.32 ± 0.74
Zn	261.39	175.07 ± 24.66	183.57 ± 30.72

### Retention and transfer abilities of the potentially toxic elements within test plants

Shoot PTE contents under all treatments were less than 30.00% of the overall bioaccumulated PTE in each plant. Both plants' minimal shoot PTE accumulation was proportional to their corresponding root storage. This low storage of bioaccumulated PTE in the above-ground parts of both plants consistently resulted in lower transfer factors (1 < TF) for both plants and a high bioconcentration factor (BCF) as shown in Table 4.

**Table 4:** Estimated phytoremediation indices of *Jatropha curcas* and *Manihot esculenta*

Potentially Toxic Element (PTE)	Jatropha curcas bioaccumulation			Manihot esculenta bioaccumulation		
	TF	BCF (mg/kg)	BAF (mg/kg)	TF	BCF (mg/kg)	BAF (mg/kg)
Cd	0.26	2.29	0.60	0.23	3.95	0.92
As	0.26	1.94	0.50	0.26	1.27	0.33
Pb	0.27	2.33	0.64	0.14	4.72	0.67
Hg	0.45	2.47	1.11	0.23	4.30	1.01
Zn	0.16	2.44	0.40	0.18	2.90	0.51

*(BCF* > *1 indicates high root PTE accumulations potentials, BAF values* > *1 indicates high shoot PTE accumulations potentials and TF* < *1 indicates low potentials for PTE transport to above ground parts)*

### Influence of soil amendments on plant biomass production

**Fig. 3:**
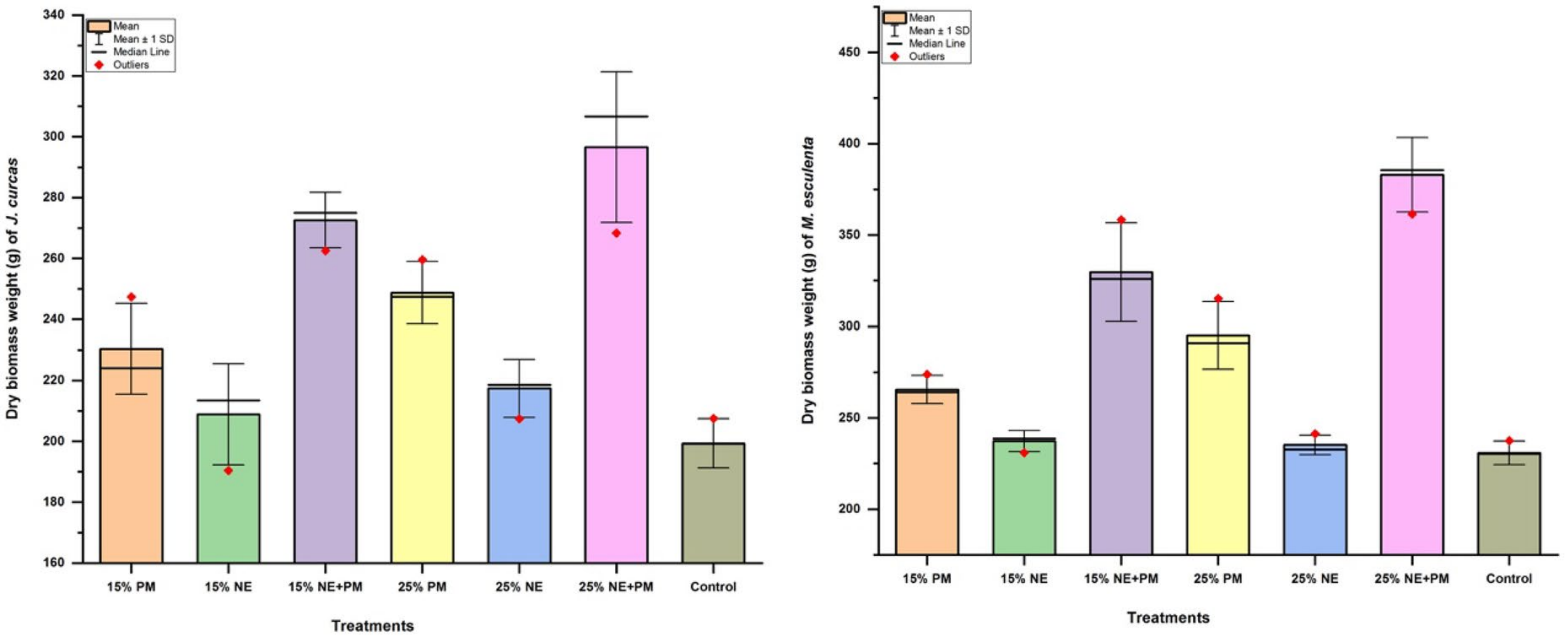
Effect of applied amendments on the biomass output of test plants

All applied organic amendments increased the biomass output of both plant species at varying levels. Their respective dry biomass weight (average) followed the order; control < 15% NE < 25% NE < 15% PM < 25% PM < 15% Neem + PM < 25% Neem + PM for both plants. Thus, when compared with their control treatments, an increase in biomass in the range of 4.60– 33.00% for *J. curcas* and 2.20– 42.00% for *M. esculenta* was achieved (Fig. 3). Additionally, control treatments produced about 6.00– 23.00% more fresh root length than those treated with organic amendments. Signs of necrosis were largely observed in the leaves of the control plants and intermittently in 15% NE and 25% NE treated plants. These, however, were reduced marginally for a few days after each watering regime.

## Discussion

### Uptake potentials of PTE in soils by *Jatropha curcas* and *Manihot esculenta*

The ability of plant roots to release acidic exudates (phytosiderophores) and bacteria have been found to assist them in the adsorption of potentially toxic elements (PTEs) from their growth media (Okoroafor et al., 2022b). Even so, different plants are noted to have different adaptation abilities to soil contaminants, hence their varying suitability for phytoremediation. This makes screening for efficient accumulator plants a key managerial decision to ensure the success of sustainable soil cleaning approaches. Despite this, there exist difficulties to pinpoint the genes responsible for a given adaptation as plants are known to have intrinsic genetic systems that regulate metal tolerance, accumulation, and survival (Okoroafor et al., 2022a, 2022c). In this present study, the similarities (*p* > 0.05) in PTE uptake capacity observed for both *Jatropha curcas* and *Manihot esculenta* could be due to a presence of a common adaptation and transport mechanism amongst them. Thus, owing to both plants being of the same family, Euphobiaceace, a physiological pathway might have influenced their akin PTE uptake (Mensah et al., 2022).

Manihot esculenta is a root storage plant widely grown for its high starch contents. The roots account for over 60% of its total biomass weight and can extend in the soil up to 1.00 m relatively faster than *J. curcas*. It is believed that these features might have aided the plant to take up more PTE further away than the *J. curcas* within the study period (Kos et al., 2003; Saifullah et al., 2009). Hence its superior performance is based on average PTE uptake. Nevertheless, the high multi-contaminant uptake potentials of both plants in their ground structures other than aerial biomass was an exhibition of excluder plant properties (Mehes et al., 2013). Plants with such properties have been profiled as suitable for the phytostabilization of contaminated media due to their adaptive strategies for survival (Marrugo-Negrete et al., 2020). The performances of both *J. curcas* and *M. esculenta* can be likened to accumulator plant species including *Populus tremula, Tanacetum vulgare, Lotus corniculatus* and *Agrostis capillaris* which are recommended hyperaccumulators for remediating PTEs such as Cd, Zn, As, and Pb from former mine sites in Germany (Wiche et al., 2017). The contents of As and Zn removed by both *Jatropha curcas* and *Manihot esculenta* for instance were found to be about 10 folds more than that reported for native accumulator plants like *Pityrogramma calomelanos*, *Chromolaena odorata*, and *Pueraria montana* found in Ghana (Kobina et al., 2021; Mensah et al., 2020a). This highlights the superior potential of the test plants investigated even though, *Pteris vittata* has been documented for accumulating soil As of about 2,500 mg/kg (Mensah, 2021; Zhao et al., 2002).

### Effects of applied organic amendments on PTE uptake

According to Kos et al., (2003), the geochemical behaviour and interaction of elements in soils can strongly influence how much PTEs can be mobilised for absorption, translocated and/ or stored by plants. The individual effects of poultry manure (Christian et al., 2021) and biochar (Amirahmadi et al., 2020; Lu et al., 2017) on soil PTE have been widely investigated. Thus, in this study, soil PTEs might have formed complexes with the applied organic amendments which limited their solubility, mobility, and availability for plant uptake (Kos et al., 2003; Okoroafor et al., 2022a, 2022b, 2022c). This means the application of 25% PM + NE which caused the least soil PTE- plant transfer might have provided superior conditions to efficiently immobilize trace elements in the soils than the remaining amendment combinations or separately. Previous studies established the reduction in mobility of Cr and Zn in soils by 70% after the addition of 20% (w/w) of poultry manure (Christian et al., 2021; Lu et al., 2017). The results of this study, therefore, confirm the immobility effect of biochar on soil PTE.

Biochar applications are well documented to enhance the adsorption abilities of cations by increasing the negative charges of soils. Also, their porousness and high surface area enable them to form complexes with organic carbon. Similarly, the mineralization of poultry manure can release cations, which increases soil pH. These phenomena might be the reason for the increased soil pH from 4.8 to 6.7- 8.3 as these pathways are notable reasons for their abilities to increase the pH of growth media and subsequently immobilize soil contaminants meant for plant uptake (Amirahmadi et al., 2020; Lu et al., 2017). Especially as the bioavailability of PTEs for plant uptake is largely regulated by the pH of the growth media and its redox potentials (Lu et al., 2017). Thus, the added organic amendments in this study created plant-soil-amendment interactions that were effective in the significant reduction in residual PTE in plants through pH alterations and the formation of complexes.

Apart from low pH, high temperatures have been established as abiotic conditions that enhance faster metabolic and biochemical processes for PTEs bioaccumulation in plant (Kumar et al., 2018). This means the generally low acidity of the background soil and the high average temperatures (38.5 ˚C) that prevailed during plant growth might have influenced the solubility, mobility, and bioaccumulation of more PTEs in the controlled plants investigated (Wang, 2016).

### Effects of applied organic amendments on plant’s biomass production

The different plant species used in this study differed in their growth habits and total biomass production due to the differences in the plant morphology. Test plants' adaptation strategies under control treatments to growth stressors (low soil nutrients, weed competitions, low soil water and multi-contaminant presence) could be attributed to the 22% more root length than those with amendment addition. The reason could be that since the growth media of the control treatment was nutrient deficient, the roots of the plants might have plants extended deeper in their growth media in the search for growth substances. Even that, long roots did not yield high biomass due to the reported 10- 25% dry biomass losses observed in the control treatments.

Incorporating organic amendments can increase soil pH, CEC, SOC, and micro and macro nutrients of soils. Instances, where animal dung boosted the organic C, accessible P, exchangeable cations, and phosphorus of soils, exist (Amirahmadi et al., 2020; Soremi et al., 2017). Also, reports of how biochar significantly increased plant growth parameters such as height, leaves, and girth after its addition to soils have been reported (Lu et al., 2017). As expected, the applied organic amendments were good sources of plant nutrients for growth and development (Table 2). The observed 10- 25% increase in dry biomass weight of amended treatments could therefore be attributed to the addition of poultry manure and neem biochar in soils which improved soil nutrition. Even that, the combined effects of both amendments created better soil conditions required for improved performances in plant growth than their individual applications. It could, therefore, be deduced that the prevailing growth stressors (low plant available nutrients, high temperatures and/ or possible reactions to elemental toxicities in the soil, weeds competitions) contributed to the observed necrosis, limited growth, and subsequent low biomass outputs in the control plants (Odoh et al., 2017; Mensah et al., 2022b; Sine et al., 2020; Singh et al., 2014). Cases, where soil acidity, water stress, and high PTE contents resulted in up to 50% plant biomass loss in *Vigna unguiculata* and *Sedum alfredii*, have been reported (Marrugo-Negrete et al., 2020; Rosa et al., 2021; Tian et al., 2022). The understandings of these scenarios indicate that even though hardy plants may survive growth stressors, adequate water and nutrient supply are essential for optimal phytoremediation performances (Parisa et al., 2010; Sine et al., 2020).

### Uptake, retention, and transfer of potentially toxic elements within plants

The low bioaccumulation factor (BAF) and transfer factor (TF) values for *J. curcas* and *M. esculenta* proved that they could be best utilized as phytostabilisers (Mensah et al., 2022b). Their soil cleaning potentials based on bioconcentration factors in this study were found to be superior to some recommended hyperaccumulators (eg., *Pityrogramma calomelanos*, *Chromolaena odorata*, and *Pueraria montana, Oenothera biennis, Pinus radiata, H. sativum and Commelina communis*) reported across the globe (Bansah & Addo, 2016; Burachevskaya et al., 2021; Mensah, 2021; Mensah et al., 2022a; Norini et al., 2019). Using root-storing contaminants plants like those reported in this study presents multiple benefits of limiting the transfer of pollutants to primary consumers and subsequent food chain contaminations (Mahar et al., 2016). Their improved roots and biomass output can further control soil erosion and help recolonize vital microorganism populations in soils lost to mining (Bartucca et al., 2022).

## Conclusion

Using *Jatropha curcas* and *Manihot esculenta* was effective in removing multi-contaminants such as Cd, Pb, As, Hg and Zn by up to 60% from soils. Storage of absorbed contaminants occurred largely in their roots culminating in a bio-concentration factor (BCF) greater than 1. In this case, such plants may be better utilized as phytostabilizers.

The addition of biochar, poultry manure or their combination at different rates was effective in immobilizing significant portions of the target elements in the soil. This occurred by increasing soil pH from 4.8 to 6.7- 8.3 and forming complexes with the organic carbon contents. Additionally, they helped with the improvement of soil nutrients for plant growth and development leading to high biomass productions.

Using phytostabilizer plants can help reduce toxicities to primary consumers and associated food chain contaminations. Therefore, opportunities for technological improvement to help optimize their large-scale applicability for soil remediation projects are encouraged. Also, efficient measures are further encouraged to help utilize the safe reuse of the contaminated biomass generated during phytoremediation. For example, biodiesel can be generated from *Jatropha curcas* seeds, whilst biomass from *Manihot esculenta* can be used for biogas production to complement community electricity supplies.

## Data Availability

All data supporting the findings of this study in its current form are available within the paper.
